# A new 3D, microfluidic-oriented, multi-functional, and highly stretchable soft wearable sensor

**DOI:** 10.1038/s41598-022-25048-x

**Published:** 2022-11-28

**Authors:** Mohsen Annabestani, Pouria Esmaeili-Dokht, Ali Olyanasab, Nooshin Orouji, Zeynab Alipour, Mohammad Hossein Sayad, Kimia Rajabi, Barbara Mazzolai, Mehdi Fardmanesh

**Affiliations:** 1 Department of Electrical Engineering, Sharif University of Technology, Tehran, Iran; 2 School of Mechanical Engineering, University of Tehran, Tehran, Iran; 3 Bioinspired Soft Robotics Lab, Istituto Italiano di Tecnologia, 16163 Genoa, GE Italy

**Keywords:** Biomedical engineering, Electrical and electronic engineering

## Abstract

Increasing demand for wearable devices has resulted in the development of soft sensors; however, an excellent soft sensor for measuring stretch, twist, and pressure simultaneously has not been proposed yet. This paper presents a novel, fully 3D, microfluidic-oriented, gel-based, and highly stretchable resistive soft sensor. The proposed sensor is multi-functional and could be used to measure stretch, twist, and pressure, which is the potential of using a fully 3D structure in the sensor. Unlike previous methods, in which almost all of them used EGaIn as the conductive material, in this case, we used a low-cost, safe (biocompatible), and ubiquitous conductive gel instead. To show the functionality of the proposed sensor, FEM simulations and a set of designed experiments were done, which show linear (99%), accurate (> 94.9%), and durable (tested for a whole of four hours) response of the proposed sensor. Then, the sensor was put through its paces on a female test subject’s knee, elbow, and wrist to show the potential application of the sensor as a body motion sensor. Also, a fully 3D active foot insole was developed, fabricated, and evaluated to evaluate the pressure functionality of the sensor. The result shows good discrimination and pressure measurement for different foot sole areas. The proposed sensor has the potential to be used in real-world applications like rehabilitation, wearable devices, soft robotics, smart clothing, gait analysis, AR/VR, etc.

## Introduction

Recently research on soft sensors was increased due to the need for proper wearable devices. Some trending wearable devices are smart fabrics^1,2^ and Smart Electro-Clothing Systems^3^, sweat sensors^4,5^, artificial skin^6^, health monitoring systems^7–9^, and motion capturing devices^10^. Another field that has attracted lots of attention is the employment of soft sensors for the need for virtual reality (VR) and augmented reality (AR) systems^11^. There are many applications in the field of soft sensors, including immersive entertainment, teleoperation, or even physical therapy^12^. Being flexible is one of the significant parameters in wearable devices. Therefore, the field of wearable devices is turning into using soft sensors. There are several types of soft sensors, including capacitive soft sensors^13,14^, electro-active polymer-based soft sensors^15–18^, and resistive soft sensors. Capacitive sensors have shown the ability to detect touch^19–21^, stretch^22–24^, or touch and stretch without the ability to distinguish between these two^25^. This type of sensor has many advantages like accuracy and easy sensor replacement but also exhibits non-linear behavior; it is oversensitive and requires a numerical method to identify the unknown capacitances^26^. The other type of soft sensor is the electro-active polymer-based (EAP-based) soft sensor, which is divided into two classes depending on the primary type of their charge carrier: ionic EAPs^17,18,27–29^ and electronic EAPs^30^. Despite having low weight, high sensitivity, biocompatibility, and significant produced signal, these sensors have some drawbacks, like being expensive, slow response, sensitivity to moisture and temperature during operation, and their function is also limited to low temperature due to liquid electrolyte^30^. The third type of soft sensor is the resistive soft sensor, which is used in this paper. It exhibits a linear response and could be fabricated using low-cost materials that allow it to be mass-produced. However, it lacks noise immunity and has low-frequency responsivity. One approach to designing a resistive soft sensor is the microfluidic-oriented approach which is the one used in this paper.

Various fabrication methods of resistive-based, microfluidic-oriented soft sensors have also been proposed. Yong-Lae Park et al. have previously reported the fabrication of a hyperelastic pressure-sensing resistive-based device by embedding silicone rubber with microchannels of conductive liquid eutectic gallium–indium^31^. Yong-Lae Park et al. also designed and fabricated a soft artificial skin using the previous method but a different channel design^32^. Mengüḉ et al. have proposed a design for a soft wearable motion-sensing suit for the application of lower limb biomechanics measurements. The proposed sensors consist of silicone elastomer with embedded microchannels filled with conductive liquid, EgaIn^33^. Mengüḉ et al. also used the same method in another paper to design wearable soft sensing suits for human gait measurement^34^. Vogt et al. used the same method and material but exhibited a novel design^35^. The mentioned paper proposed a soft multi-axis force sensor that was capable of measuring normal and in-plane shear forces. Joseph T. Muth et al. have reported a new method, known as embedded 3D printing (e-3DP), which involves extruding a viscoelastic ink through a deposition nozzle directly into an elastomeric reservoir. They also used carbon conductive grease as the functional ink for patterning sensing elements within the 3D printed device^36^. Seokbeom Kim et al. developed a stretching and twisting sensor that employs a printing method to implement the desired pattern with EgaIn^37^.

As discussed, almost all the papers reviewed before have used EGaIn as the conductive material filling the microfluidic channels^38^. EGaIn shows relatively low toxicity, but this cannot be concluded that both ions are non-toxic. Therefore, EGaIn is reasonably safe to use in an aqueous environment, but it should be cautiously handled to stay safe when any mechanical agitation is applied^39^. EGaIn is a liquid metal with high conductivity. However, the high conductivity of this liquid metal makes the total resistance of fabricated sensors significantly low (about 1Ω and lower), which requires complicated circuits to measure the resistance and any change in the output signal. Unlike sensors made of EGaIn, the sensors utilizing conductive gel have a resistance of about a few kΩ which makes it easy to measure, and though it requires a less complicated circuit. Table 1 shows characteristics of EGaIn and conductive gel.

**Table 1:** Comparison between characteristics of EGaIn and conductive gel.

	Conductive gel	EGaIn
Compound	Water, EDTA, Imidazolinyl Urea, etc	Ga 75.5%/In 24.5%
State of matter	Gel	Liquid
Viscosity	130,000–185,000 cps	Low-viscosity (N/A)
Color	Translucent	Silver
Resistivity	10^3^ Ω mm	2.94 × 10^−4^ Ω mm
Producer	Aquasonic	Sigma-Aldrich
Price	~ 0.5 €/100gr (Super Cheap)	~ 2300 €/100 gr (super expensive)

Even so, EGaIn is very expensive compared to conductive gel, which increases the cost of fabricated soft sensors and makes it difficult to mass-produce. Moreover, by using EGaIn as a conductive material in microfluidic channels, after a while, it leaves sediment a notable amount of sediment. On the other hand, there was no sediment left while using conductive gel.

To overcome these issues, we used conductive gel (known as ultrasound gel), which is non-toxic, durable, and inexpensive. Inspired by reviewed strategies, we proposed a novel method for fabricating microfluidic-oriented resistive soft sensors, enabling us to implement any desired 3D pattern. By making 3D sensors, we can measure stretch, twist, and pressure simultaneously with a single sensor. Researchers have recently attempted to propose methods for fabricating 3D soft sensors. Yue Zhao et al. have proposed a method for fabricating conductive 3D metal-rubber composites for stretchable electronic applications^40^.

Furthermore, Kyung-In Jang et al. introduced a three-dimensional network design comprised of helical coils^41^. Aw et al. Investigated an elastomer-based piezoresistive utilizing Ecoflex and carbon black^42^. Olson et al. proposed a novel conductive gel and PDMS composite material as a potential stretchable sensor^43^. Shin et al. developed a soft data glove using Ecoflex and EGaIn for hand gesture recognition^44^.

Employing the mentioned methods, we cannot fabricate any desired pattern. This paper presents a novel method for fabricating any desired 3D pattern, and FEM simulations are performed to prove the concept. After that, three different soft sensors are fabricated using the proposed method and then experimented with. Afterward, one of the sensors is used in a real-world application. Finally, a fully 3D foot insole is fabricated and tested.

## Proposed method

**Figure 1:**
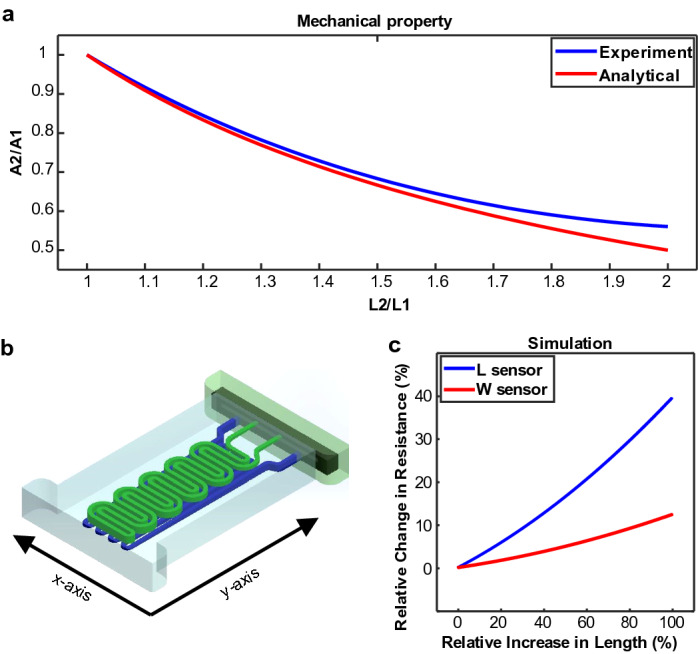
Sensor design (Type-2) and simulation results. (**a**) Comparison between analytical and simulation data of the mechanical properties, (**b**) the schematics of the sensor used in the simulations, (**c**) Simulated sensors’ characteristics.

As stated previously, the simple nature of resistive sensors enables us to have a definite prediction of their behavior. Theoretically, if a conductive object's shape changes, its electrical resistance will also change. However, if the conductive material has a variable geometry in all axis components, the electrical resistance would be affected by external stimulations in any axis. Toward these goals, first, we need a highly soft conductive material, and second, a 3D structure is required for the conductive material to have three variable axis components. For this aim, we have proposed a technique to fabricate 3D microfluidic channels into highly stretchable materials like silicon rubber to achieve variable elements on all axis. Suppose we fill the fabricated 3D microchannel with a conductive gel (which is called the active part). In that case, we will have a soft active part with a stable but deformable 3D structure that could respond to different stimulations such as stretch, twist, and pressure as a highly stretchable soft sensor. In (Fig. 1a), an example of the proposed soft sensor (Type-1) is illustrated, which has two individual active parts (W-Sensor and L-Sensor) for the measurement of transverse and longitudinal mechanical stimulations. We have proposed two other structures, described in the following sections.

As the starting point, we pick Pouillet’s law and gradually determine the sensor's response well enough. Finally, by a finite element model (FEM), we will show that the proposed idea works and has reliable fidelity to reality.

### Theoretical justification

As shown in (Fig. 1a), the designed sensor has two active parts. In the rest of the analysis, the L-sensor, which has long components along the y-axis, has been used. Furthermore, we can apply the same principle to the other sensors due to the generalizable argument behind the justification. The active parts are filled with a conductive gel that can be interpreted by its resistivity $\rho$. We can divide the L-sensor into several elements as $n1$ identical components in the $y$-direction with the length of $L1$ and $n2$ identical components in the $x$-direction with the length of $L2$. Now that we have divided the problem into its components, we can approach it more directly. We can assume that $n1$ $y$-direction components and $n2$ $x$-direction components are connected in series form, and so based on Pouillet's law, the net electrical resistance of all components ($R_{T}$) of L-Sensor is as follows:

1 $$R_{T} = n_{1} \rho_{1} \frac{{L_{1} }}{{A_{1} }} + n_{2} \rho_{2} \frac{{L_{2} }}{{A_{2} }}.$$

The channel is fixed in cross-section throughout the sensor for the proposed designs. Also, the conductive gel is uniformly distributed within the channel, so $\rho = \rho_{1} = \rho_{2}$ and $A_{1} = A_{2} = t \times w$, and the following equation will be obtained:

2 $$R_{T} = n_{1} \rho \frac{{L_{1} }}{t \times w} + n_{2} \rho \frac{{L_{2} }}{t \times w}$$

where *t* and *w* are thickness and width, respectively. We can assume that for a fixed volume, the $\alpha$ times elongation in any direction leads to an $\alpha$ times cross-section shrinkage and $\sqrt \alpha$ times *t* and *w* shrinkage, which can be formulated as follows:

$$Fixed\;volume = L \times A \to if\;L^{\prime } = \alpha L \to A^{\prime } = \frac{1}{\alpha }A$$

If the applied force is uniform, we have:

3 $$t^{\prime } = \frac{1}{\sqrt \alpha }t,\;w^{\prime } = \frac{1}{\sqrt \alpha }w$$

Any stretch along the y-direction will result in the same situation for the y-oriented components, although there is more complexity for the x-oriented ones. If they were situated in the middle of the sensor, we could confidently assume that the channel's width would stretch with the same factor, which is not the case here. The amount of width elongation for those channels depends on one more factor: the properties of the flexible material. First, we assume that the width of the x-oriented channels will stretch by a factor of $\beta$, so Eq. (2) becomes:

4 $$R_{T}^{\prime } = n_{1} \rho \frac{{\alpha L_{1} }}{{\frac{1}{\alpha }t \times w}} + n_{2} \rho \frac{{\frac{1}{\sqrt \beta }L_{2} }}{{\beta t \times \frac{1}{\sqrt \beta }w}}$$

where $n_{2} = n_{1} - 1$ and consequently, we have:

5 $$R_{T}^{\prime } = \frac{{n_{1} \rho L_{1} }}{t \times w} \left( {\alpha^{2} + \frac{1}{\beta } \times \frac{{L_{2} }}{{L_{1} }} } \right) - \frac{1}{\beta } \times \frac{{L_{2} }}{{L_{1} }}$$

In the L-Sensor, the length of $L_{2}$ is far smaller than of $L_{1}$ (50 times). Moreover, the $\beta$ is approximately $\alpha ,$ as stated before, so it is safe to assume a change in $L_{2}$ would be negligible. Hence the approximated form of Eq. (5) is obtained as Eq. (6):

6 $$R_{T}^{\prime } = \frac{{n_{1} \rho L_{1} }}{t \times w} \alpha^{2}$$

It is inferred from Eq. (6) that the output signal of the L-Sensor is a 2nd-degree function of the $\alpha$ stretch factor. We can apply the same approach to determine the electrical resistance of other presented sensors. However, we should consider that we cannot have the last assumption of negligible length ratios in every sensor. So, the relation will deviate more from Eq. (6).

The dual-stage sensor in (Fig. 1a) was simulated in COMSOL to test our analysis. In order to achieve a more realistic response from the simulation, we conducted a simple experiment to determine the relationship between the increased length and the shrinking cross-section. As shown in (Fig. 1c) the experimental data follows the theoretical assumption. The mentioned experimental data were used to generate a set of different thicknesses, lengths, and widths and fed to the COMSOL as parameters. Then, resistance was calculated in each set of parameters that determined the sensor’s mechanical state. Finally, the results were fed to MATLAB for post-processing and ratio calculations. It can be seen from (Fig. 1b) that the mentioned sensors have a second-degree behavior which will translate to a more linear characteristic due to the small amount of displacement in real-life applications. Although, it is evident that the W-sensor has more non-linearity due to the more clear non-linear term (Eq. 5).

**Figure 2:**
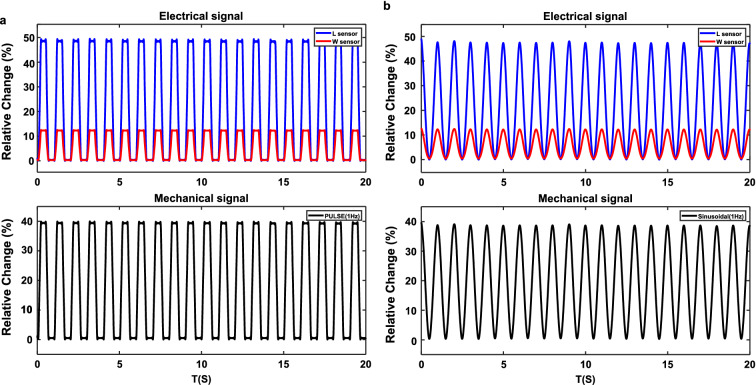
Simulation output of (**a**) pulse shape and (**b**) sinusoidal mechanical stimulation.

At this stage, a sinusoidal and Pulse shape mechanical wave was generated to show the L and W sensor’s functionality (Fig. 2). These examples show that the output electrical signals of the proposed sensors are highly correlated to the input mechanical stimulations, so the sensor can work linearly. In the following sections, various experimental tests will be done to validate the proposed sensor’s response.

### Ethical considerations in the proposed method

In this study, there were done no experiments on human organs/tissue and the proposed sensor has had no physical contact with the human skin. We did NOT expose the subject to treatment or have a protocol to follow certain behavior at all. Moreover, we did not have a population study and we only tested it on one of us (with her consent) to check the feasibility of our study on gait analysis applications. For potential future analysis on studying the performance of our proposed method on a larger group of individuals, the required ethical agreement will be provided.

## Fabrication procedure and experimental data acquisition

**Figure 3:**
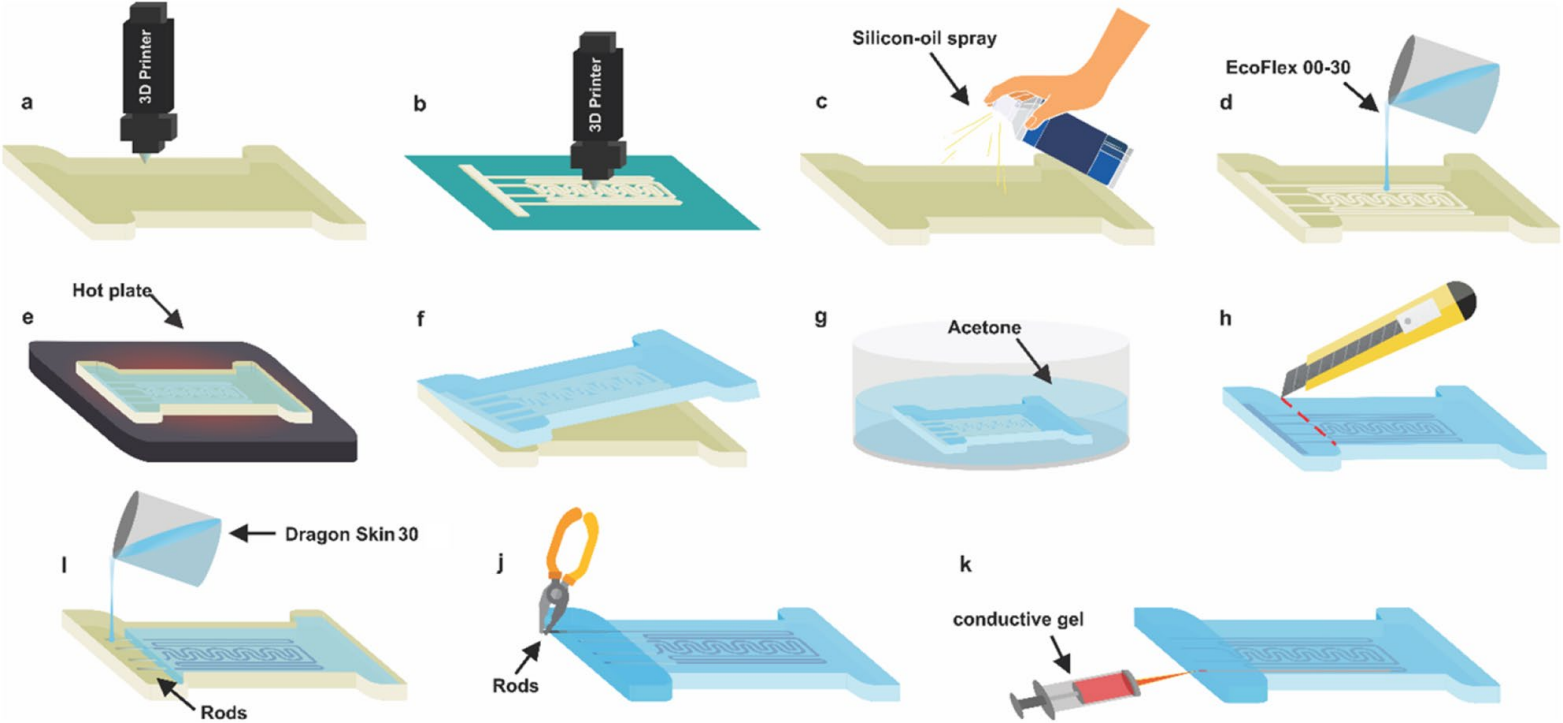
Fabrication process (**a**) 3D printing of the mold with ABS. (**b**) 3D printing the scaffold with ABS. (**c**) Spraying silicon oil on the mold, (**d**) pouring the Ecoflex 00-30 into the mold. (**e**) Curing Ecoflex 00-30: heated to 120° for 20 min. (f) peeling off the chip from the mold. (**g**) Dipping the chip into the acetone. (**h**) Cropping the clamp region. (**i**) Filling clamp region with Dragon Skin 30 (room-temperature-vulcanizing) silicone rubber. (**j**) Extracting the rods from the chip. (**k**) Filling the channel with conductive gel (a combination of water and propylene glycol).

The fabrication steps for the proposed sensor are represented in (Fig. 3). First, the mold and scaffold are prepared by 3D printing using an FDM 3D printer. Acrylonitrile Butadiene Styrene (ABS) was used as printer's filament which is a common thermoplastic polymer. Also, to prevent the soft material from excessive adhesiveness to the mold, the silicone-oil spray is used as a lubricant so that the sensor can be peeled off easily from the mold. After that, the printed scaffold pattern is inserted into the mold. The Ecoflex 00-30, a type of silicon rubber, liquid elastomer, is used as the soft material. After preparation of Ecoflex and placing it in the desiccator to expel remaining bubbles, it is poured into the mold. Then it is placed on a hot plate and heated to 120° until Ecoflex is cured. The curing time is about 20 min and depends on the thickness of the sensor. After peeling off the chip from the mold, it will be submerged in acetone. By doing this, the acetone vapor dissolves the ABS, and the 3D microchannels are created into the Ecoflex.

In this step, the sensor is ready and can be filled with conductive gel, but because of the high elongation of the Ecoflex, the solid contact wires may not stay fixed in the channel and will cause an unstable response. Thereby we have used another material with less elongation for the clamp region.

**Figure 4:**
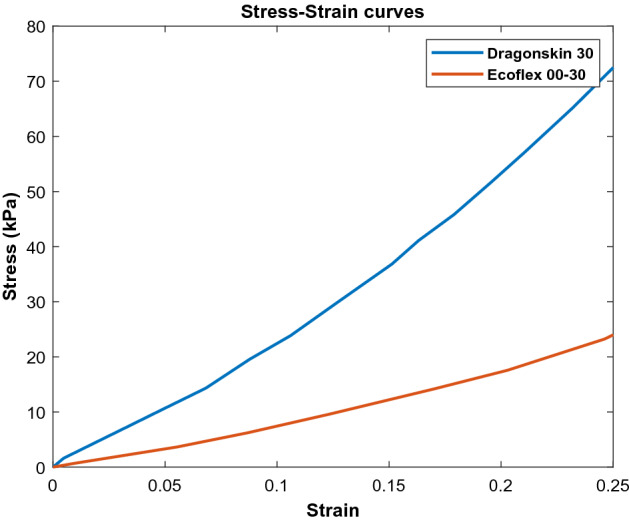
Stress–strain curve of Ecoflex 00-30 and Dragon skin 30.

Table 2 shows the characteristics of both Ecoflex 00-30 and Dragon Skin 30. As shown in the table, Ecoflex has higher elongation (900%) and is used in the active area of the soft sensor to provide highly-stretchability. On the other side, Dragon Skin 30 has higher shore hardness and lower elongation, though it is used for the clamp part of the soft sensor to have more sustainable electrodes. Figure 4 shows the stress–strain curve of Ecoflex 00-30 and Dragon Skin 30 which was obtained from experiments done by sparks et al.^45^.

**Table 2:** Comparison between characteristics of Ecoflex 00-30 and Dragon skin 30.

Characteristic	Ecoflex 00-30	Dragon skin 30
Viscosity	3000 cps	20,000 cps
Shore hardness	00-30	30 A
Tensile strength	200 psi	500 psi
100% modulus	10 psi	86 psi
Elongation	900%	364%
Producer	Smooth-on	Smooth-on

Thus, after removing ABS from the chip, the clamp region is cut, and the chip is put in the mold again. Then the clamp region is filled with Dragon Skin 30 silicon rubber. After the chip has peeled off to avoid the appearance of any unwanted bubbles in the gel, the channels are filled out using a designed syringe pump with a range of mL/min flow rate. Finally, Electrical connections, made of thin wires, will be inserted into each reservoir.

**Figure 5:**
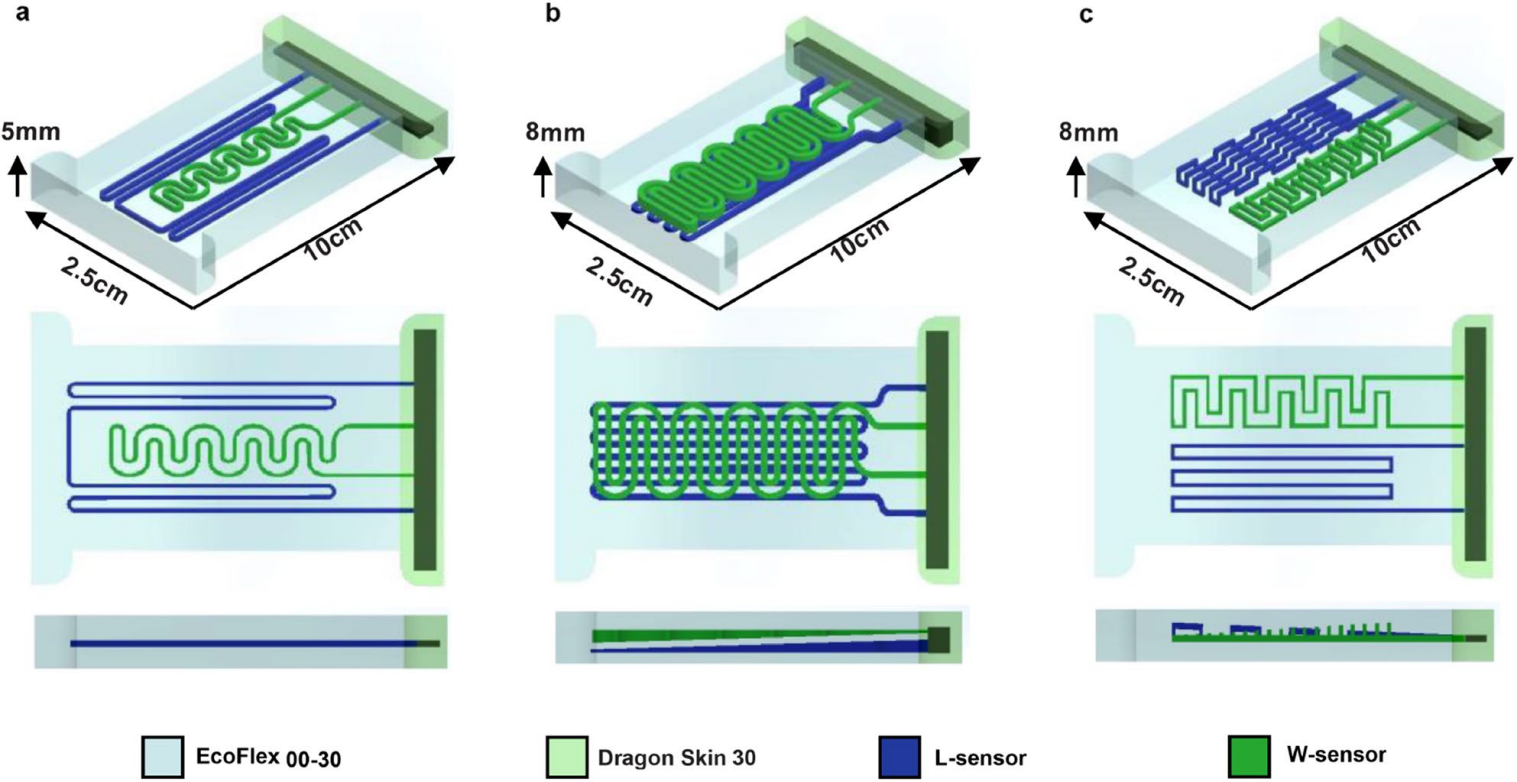
Three Sensor designs for different measuring purposes (**a**) Sensor Type-1 which is designed in planar structure (**b**) Sensor Type-2 which is designed in a double-decked structure (**c**) sensor Type-3 which is designed in fully 3D structure.

**Figure 6:**
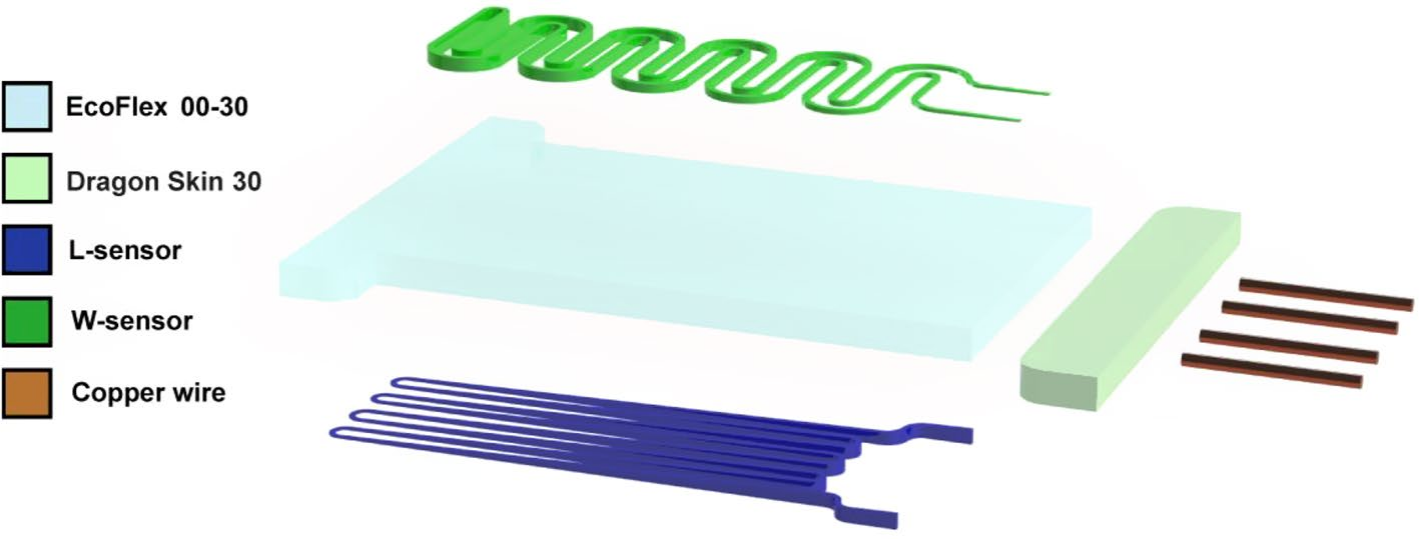
Exploded view of sensor Type-2.

Figure 5a illustrates the first sensor (Type-1), which has a planar structure. Figure 5b shows the second sensor (Type-2), which is designed in a double-stage structure in which the thickness of the channels changes linearly in opposite directions of each other, which means the thickest part of the L-Sensor is where the W-Sensor is in its thinnest part and vice versa. In other words, the sensor (Type-2) has a trapezoidal shape in the side view, designed to add the third axis component. Later it is shown that the trapezoidal design will enable the sensor to measure stimulation on the z-axis or, as we call it, the pressure. Furthermore, the third sensor (Type-3), as shown in Fig. 5c, is designed fully in 3D, with a trapezoidal shape in the side view. Figure 6 shows exploded view of the soft sensor (Type-2).

### Hardware apparatus

**Figure 7:**
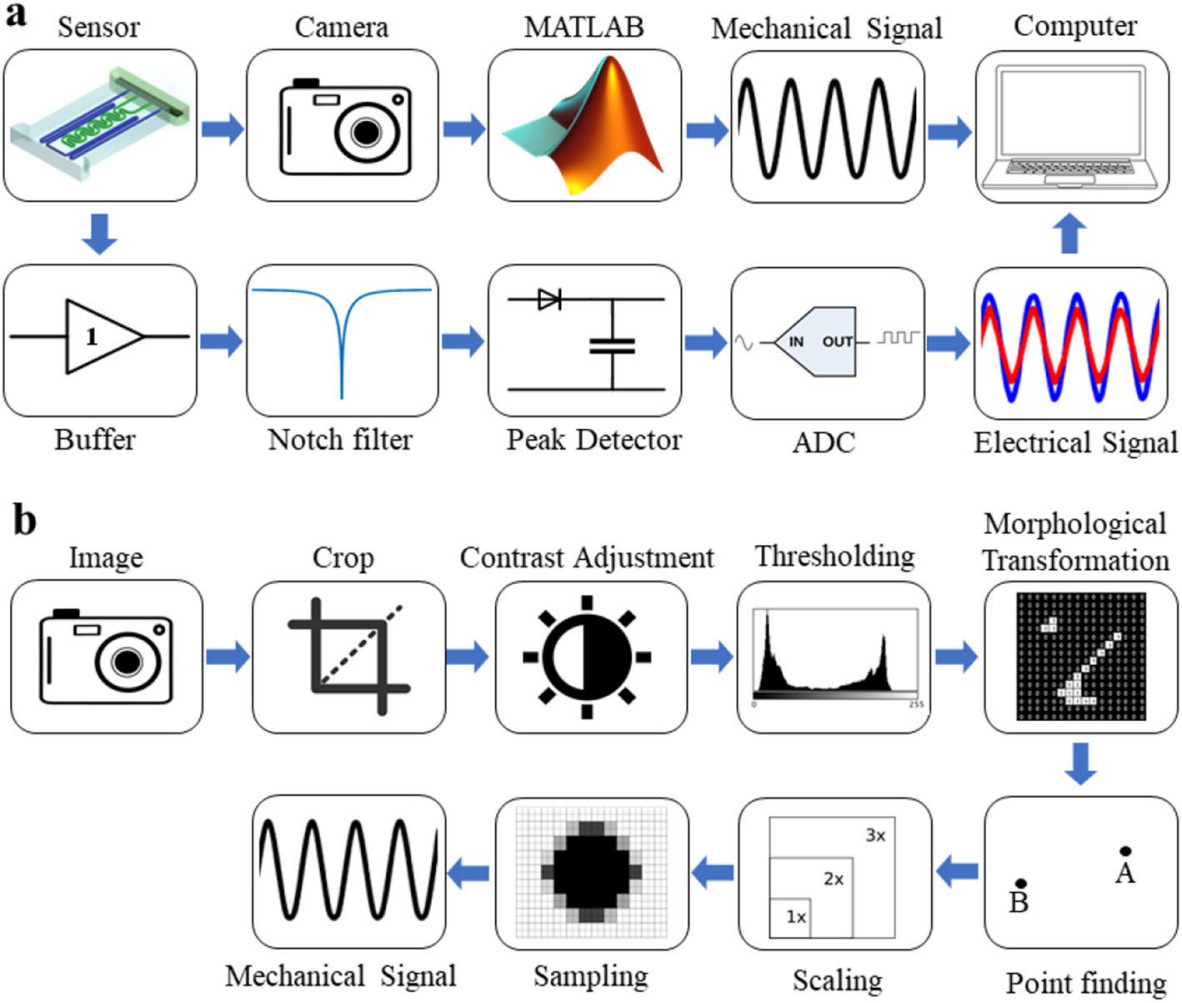
Block diagram of sensor’s testing setup. (**a**) Depicts the hardware configuration block diagram for measuring the applied mechanical signal and electrical response of the proposed sensors to the corresponding applied mechanical signals such as stretch and twist with different waveforms, (**b**) block diagram of utilizing image processing techniques to capture mechanical tension applied to the sensor.

**Figure 8:**
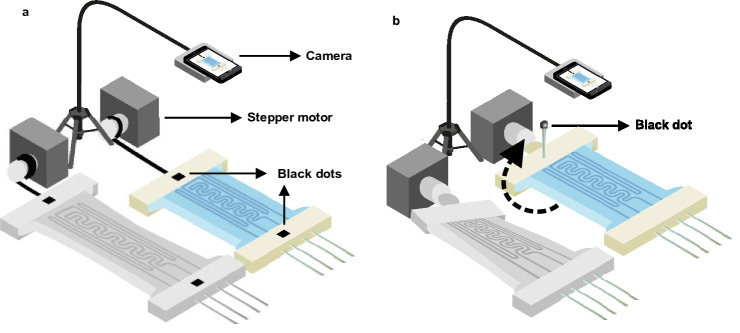
The designed setup for logging distance and angle data from (**a**) stretching and (**b**) twisting the sensor.

Figure 7A shows the hardware setup block diagram for testing the response of proposed sensors to the applied mechanical signals such as stretch and twist with different waveforms. There are two setup designs for stretching and twisting the sensors, shown in Fig. 8a, b, respectively. By using a stepper motor as a mechanical power source, the sensors were stretched or twisted between stages. Furthermore, a camera was used for capturing mechanical stress applied to the sensor using image processing methods, while an electrical setup was logging the sensor’s impedance during the test. The flowchart of the whole image processing procedure is shown in (Fig. 7b). For obtaining the exact length of the stretch and angle of twist with image processing, we need two black points on each side of the sensor for the stretch setup, shown in (Fig. 8a), and a stick with a black point on its head placed at the end of the sensor for twist setup, shown in (Fig. 8b). In each setup, the captured video was converted into an image sequence. Then the images were turned into BW images using a threshold that keeps the black points and removes other objects in the picture. In the stretch setup, the distance between two black points was calculated using morphological transforms, and the other necessary computations were done. In the setup for twist stimulation, the sensor’s twisting angle could be calculated via the position of the black point in the camera's view using the same image processing tools. Here we obtained the length of displacement for the stretch stimulation setup and the angular displacement for the twist stimulation setup. During the test, the electrical signal and the mechanical signal were synchronized by an LED. The moment the mechanical signal starts applying the stimulation, the electrical circuit captures the impedance data, and the video frames with the LED turned on are processed. Both signals could be easily coupled, and by using the coupled signal, the transform function of the following sensor could also be obtained.

During the experiment, all the sensors were subjected to mechanical signals such as Sinusoidal, Ramp, Pulse, and PRBS with stretching and twisting. Figure 8a, b show the setup for experimental tests. The mechanical signals were produced by modulating the speed of the stepper motor in different shapes of waveforms. Figure 7a depicts the block diagram of the electrical setup used to acquire and log the sensor's impedance. First, an AC signal was applied to an AC potential divider consisting of the sensor and a Resistor. Then, to eliminate the electrical loading problem, the potential divider's output was buffered and fed to a 50 Hz Notch filter to reject city power interference. After that, the output was a clear AC waveform. Because the change in the sensor's impedance would lead to a change in the amplitude of the AC waveform, a peak detector was used to obtain the amplitude of the signal. In the end, an Analog-to-Digital Converter was used to acquire the analog signal and send it to the computer by serial communication (UART). An STM32F103 microcontroller with an integrated 12-bit ADC was used to convert the analog signal to digital and send it to the computer.

## Results and discussion

**Figure 9:**
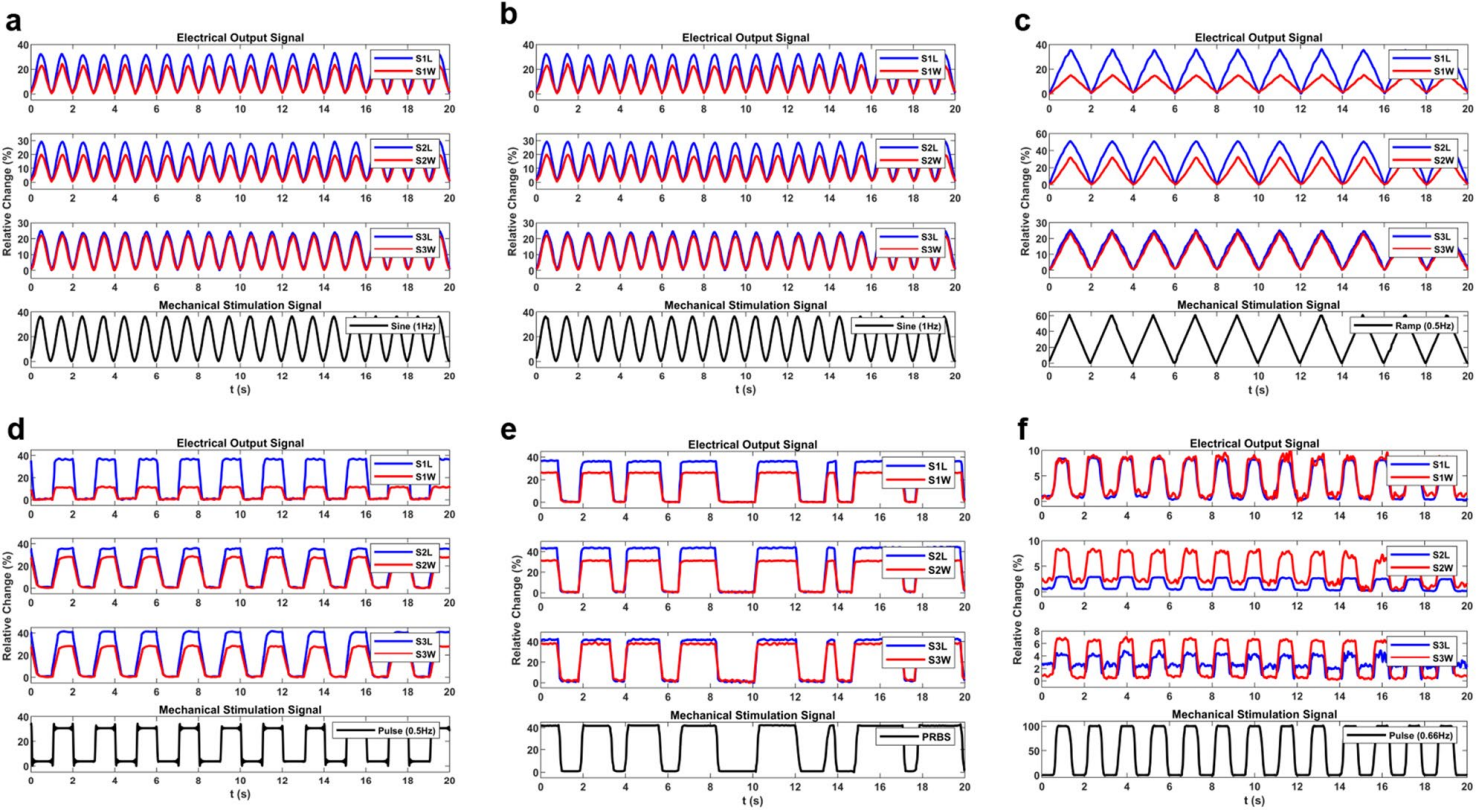
The Experimental output results of the electrical signal due to stretching with different mechanical signals of (**a**) and (**b**) sinusoidal, (**c**) ramp. (**d**) Pulse, and (**e**) PRBS mechanical signals. (**f**) Response of the soft sensors to the twist Pulse mechanical signal. *Note*: index i in SiW and SiL refers to Type-I sensors for i:1,2, and 3.

**Figure 10:**
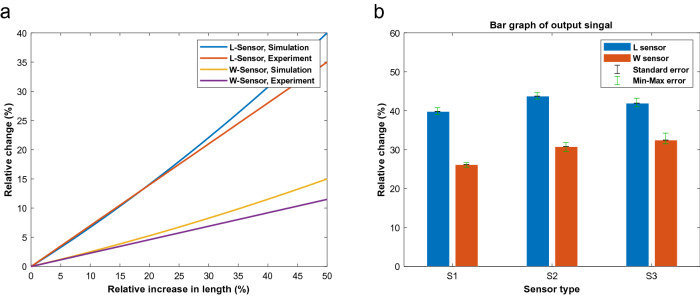
(**a**) Comparison of simulation and experimental results for sensor Type-2. (**b**) Bar graph of output signal strength including error bars.

A series of experiments have been conducted to evaluate the designed sensors and determine their real-world application capabilities. Various mechanical stimulations have been used to achieve a thorough and proper analysis of their behavior, and the variation in the impedance was recorded. Responses to the Sinusoidal and Ramp signals are shown in Fig. 9a–9c, respectively, indicating excellent performance in the devised circumstances. As shown in Fig. 9, the electrical signal is effortlessly following the input mechanical stimulation. It can be inferred that these sensors can handle high-speed stimulations as efficiently as very-slow stimulations. Figure 9f shows the experimental results of twisting the sensors from 0° to 90° and back. The output signal is well explanatory and shows a good following of the input signals. A comparison of simulation and experimental results for sensor Type-2 is shown in Fig. 10a. Clearly, there is a good correlation between experimental and simulation results for the fabricated sensor. The bar graph of signal strength in experimental tests including error bars is represented in Fig. 10b. Based on the figure, the sensor’s electrical response shows high stability and low error levels. Errors were calculated with two approaches; First, the standard error, which is defined as below:

7 $$SE = \frac{\sigma }{\sqrt n }$$

where $\sigma$ and $n$ are the sample standard deviation and number of samples, respectively. The second is the Min–Max error, which shows the maximum and minimum values of the acquired samples. Calculated Standard Error implies a maximum error of 5.08% and Min–Max error indicates a maximum error of 4.4% which leads to an accuracy of about > 94.9%.

**Figure 11:**
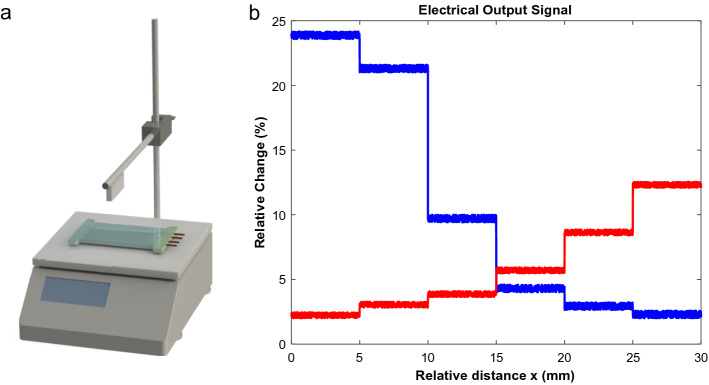
(**a**) Schematics of the setup for experimenting output response of the soft sensor to the force along Z direction. (**b**) Experiment results of electrical signal due to applying the perpendicular force of 3 N on different points of the Soft Sensor Type-2. Blue and red colors refer to L and W sensors, respectively.

The highest stretch the sensors could handle is limited by the elongation of the material used and also by the electrical circuit. Because of the high elongation of Ecoflex 00-30 (900%), we are limited by electrical circuit saturation. As shown in the figures, the electrical output response is significant, and the circuit would be saturated for higher deformations. However, a simple adjustment would make the output signal readable for up to 200% stretching. Furthermore, the lowest readable deformation is about 0.2% due to the limitation of the ADC and the effect of applied filters (such as moving average). As mentioned before, the advantage of fabricating 3D sensors is that they can be stimulated in all axes, thus measuring the change of shape in three dimensions. Previously the response in two dimensions was measured with stretching and twisting. The resistance change due to the change in the third dimension will be measured by applying pressure to the soft sensor. Six points were chosen along the longitudinal axis of the soft sensor (Type-2) with a spacing of 5 mm between them to characterize the response of the Sensor to the force along the Z direction. The experimental setup for testing the Soft Sensor is shown in (Fig. 11a). The setup contains a digital scale and a beaker stand and a piece of aluminum sheet was fixed under the horizontal bar of the stand. Afterward, a digital scale was placed near the stand, and the Soft Sensor was put on the scale. By turning the screw on the beaker stand, the height of the aluminum sheet would change, and after it reaches the surface of the Soft Sensor, it would start applying force in the Z direction on the Soft Sensor. The digital scale was used to measure the amount of force applied to each point. Though, the screw on the stand was turned until the aluminum sheet touched the surface of the Soft Sensor and started applying pressure on it until the digital scale showed the number of 300gr, which is equal to ~ 3 N force. In order to calculate the pressure applied to the soft sensor, the following equation could be used:

8 $$P = \frac{F}{A}$$

where *F* is the applied force and *A* is the area of contact. As previously stated, the applied force in this experiment is ~ 3 N. The sheet was 2 mm thick and 20 mm long, resulting in a surface area of 40 mm^2^. Consequently, each point experiences a pressure of ~ 7.5E + 4 N/m^2^ or ~ 75 kPa. While applying the pressure to each point, the output signal was acquired which is shown in (Fig. 11b). It represents the localized response of the sensor to the pressure and this remarkable response is due to the sensor's trapezoidal (3D) shape from the side view, which is the result of fabricating 3D sensors with the proposed method.

**Figure 12:**
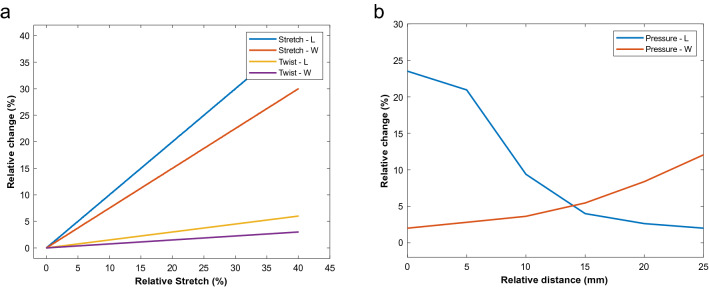
Response of the sensor Type-2 to (**a**) stretch, twist, and (**b**) pressure.

Figure 12 shows the response of the soft sensor (Type-2) to stretch, twist and pressure. By applying the stretch stimulation, the response of the L-sensor would be high, and the response of the W-sensor would be much smaller (about half). By twisting the soft sensor, the response of the W-sensor would be more significant than L-sensor. At last, applying pressure on different locations of the soft sensor shows an entirely different pattern rather than stretching and twisting. If one of the stimulations is applied, the type of stimulation would be evident by the electrical signal. The reason for the multi-functional behavior of the soft sensor is explained in the following. Because the L sensor has compartments in the longitudinal direction, stretching would cause the resistance of the L sensor to increase. However, the W sensor has longer compartments in the vertical direction, so the change in the resistance of the W sensor due to stretch would not be as much as the L sensor. Furthermore, by twisting the soft sensor, the W sensor’s resistance change will be more significant than the L sensor. Moreover, the sensors are trapezoidal in the side view, so by applying pressure, the resistance of the L and the W sensors would change differently. This was the main idea behind designing a 3D multi-functional sensor, and by the experiments, it is clear that a single soft sensor could function as stretch, twist, and pressure sensor.

For later comparison of sensitivity performances, the gauge factor is defined as below:

9 $$GF = \frac{{\Delta R/R_{0} }}{{\Delta L/L_{0} }}$$

where R_0_ is the initial resistance, and $\Delta R$ is the change in the resistance of the sensor due to stretching. $L_{0}$ is the initial length, and $\Delta L$ is the change in the length made by stretching. The gauge factor was calculated for the fabricated soft sensors, which are 3.33, 4.09, and 3.77 for soft sensors Type-1, Type-2, and Type-3, respectively. The sufficient gauge factor in addition to high stretchability makes the sensor a great candidate among the others in the literature. A comparison between the performance of the existing soft sensors has been summarized in (Table 3). It is evident that sensors with high stretchability exhibit low gauge factor and sensors with high gauge factor lack high stretchability. The paper presents a trade-off between high stretchability (200%) and a reasonable gauge factor (3.33–4.09), making it suitable for practical applications. The paper has several novelties which will be briefly explained in the following. The new method for fabricating low-cost soft sensors is the first novelty of the presented paper in which any desirable 3D pattern could be fabricated using this method. The second novelty is that the paper utilizes a low-cost and safe conductive gel instead of EGaIn, which is the first demonstration of such sensors in the literature. The third novelty arises from fabricating 3D sensors with this method which is multi-functionality as the fabricated sensor responds to stretch, twist, and pressure. While the response of the sensor to the mentioned stimulations is readable, it is also possible to discriminate which type of force is applied if the sensor is stimulated with only one type of force. By having all of these novelties in hand, our proposed sensor shows a comparable performance among the others in the literature while being low-cost, 3D, and multifunctional.

**Table 3:** Comparison the performance of the proposed soft sensor with the soft sensors in the literature.

	Lowest stretch	Highest stretch	Gauge factor	Key materials
Park et al.^32^	6%	250%	3.6–3.7	EGaIn, Ecoflex
Mengüç et al.^33^	N/A	300%	2.5	EGaIn, Ecoflex
Mengüç et al.^34^	N/A	N/A	2.2–2.5	EGaIn, Ecoflex
Vogt et al.^35^	2N	180%	N/A	EGaIn, Ecoflex
Muth et al.^36^	N/A	450%	3.8	Conductive ink, Ecoflex
Kim et al.^37^	N/A	700%	< 1	EGaIn, Ecoflex, PDMS
Liu et al.^46^	N/A	400%	0.5–1	Dragon skin, CNT
Aw et al.^42^	N/A	N/A	0.68	Carbon black, Ecoflex
Yamada et al.^47^	N/A	280%	0.82	PDMS, CNT
Olson et al.^43^	N/A	200%	< 1	PDMS, conductive gel
Cai et al.^48^	N/A	85%	35	Graphene, CNT, PDMS
Kim et al.^49^	N/A	80%	7.1	Ag, PDMS
Liu et al.^50^	N/A	700–1400%	0.005	CNT, SEBS
Li et al.^51^	N/A	120%	0.45–2.08	CNT, PDMS
Shin et al.^44^	N/A	225%	~ 0.2	Ecoflex, EGaIn
Proposed method	0.2%	200%	3.33–4.09	Ecoflex, Dragon skin, Conductive gel

### Characterization tests

**Figure 13:**
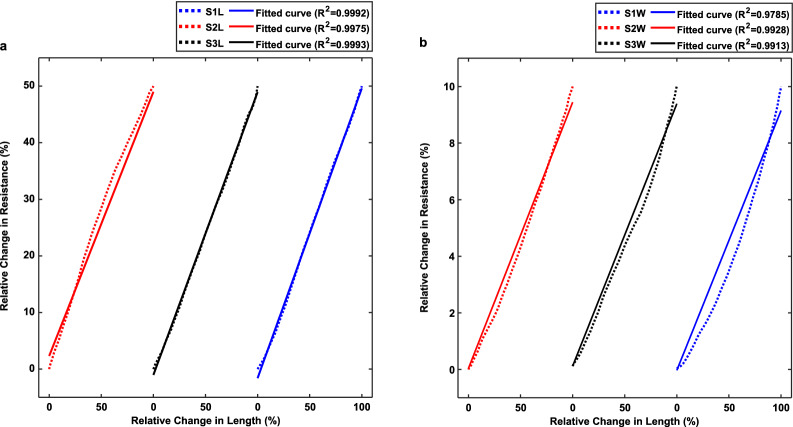
The Characterization diagrams of each of the three sensors for both (**a**) L and (**b**) W compartments. *Note*: index i in SiW and SiL refers to Type-i sensors for i: 1, 2, and 3.

As stated in the theoretical justification section, the behavior of these flexible sensors is of the second degree by making some considerations. Due to the small changes in each direction of the sensors in real-world applications, it is expected to have a near-linear behavior that is a massive advantage in data analysis and predictability. To test this hypothesis, the acquired data in the previous experiments were used to generate a characterization diagram of each of the three primary sensors for both L and W compartments, as shown in (Fig. 13). These diagrams and calculated R^2^ show that the fabricated sensors have excellent linear behavior, which was expected from previous experiments with sinusoidal and ramp mechanical signals. In addition, the sensors’ response to higher deformations would be readable but non-linear. However, utilizing polynomial estimation, curve fitting tools, and machine learning algorithms, we can determine the non-linear response of the sensors in higher deformations.

### Durability tests

**Figure 14:**
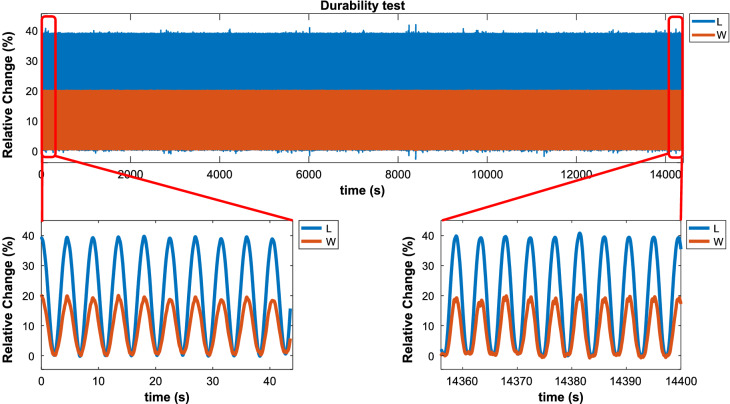
Durability test result with sinusoidal mechanical stimulation. The figure on the left is the output signal from the first seconds of the durability test and the figure on the right is the output signal in the last seconds of the durability test.

To use a sensor in practical application, besides the linearity feature, it should also have a high level of durability. The linearity of the proposed sensor was investigated in the previous section. Here, we want to show that the proposed sensor is durable enough to be used in real-world applications. To test the durability of our sensors, a 4-h experiment was designed in which the sensor (Type-1) (as an example) was subjected to stretching back and forth sinusoidally for a whole four hours. The results can be seen in (Fig. 14) which shows the sensors' performance has not seen any considerable change in this time, demonstrating that the proposed sensor is thoroughly durable.

### Real-world applications

**Figure 15:**
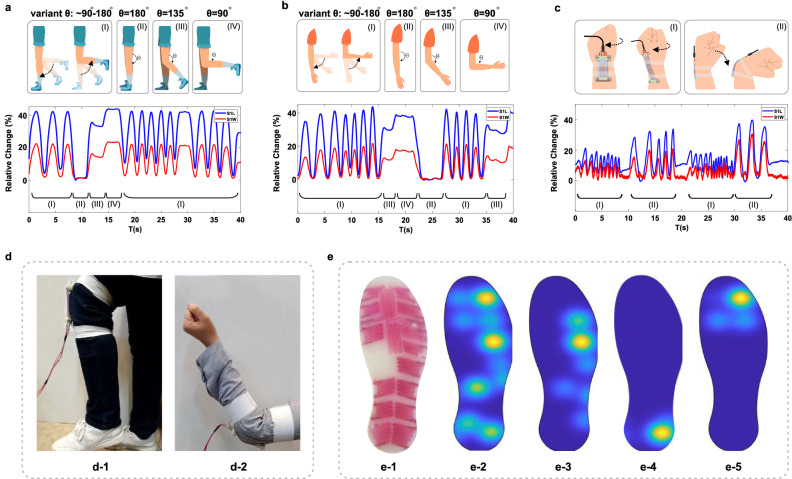
Practical results of using the sensor in real-world applications (**a**) Output signals of Knee movements such as (a-l) moving the knee sinusoidally with variant theta of 180 to 90, (a-ll) keeping the knee straight, (a-ll) bending the knee at $\theta = 135^{ \circ }$ and also (a-lV) bending the knee till $= 90^{ \circ }$. (**b**) Output signals of elbow movements such as (b-l) moving the elbow sinusoidally with variant theta of 180 to 90, (b-ll) keeping the elbow straight, (b-ll) bending the elbow at $\theta = 135^{ \circ }$ and also (b-lV) bending the elbow till $\theta = 90^{ \circ }$ (**c**) Output signals of wrist movements including (c-l) twist and (c-ll) bending motion. (**d**) actual images of testing the proposed sensor on the subject's (d-1) knee and (d-2) elbow. (**e**) the whole 3D fabricated foot insole and experimental results: (e-1) real-world image of the insole (e-2) the measured pressure gradient graph as the foot naturally applies pressure on all areas of the insole (e-3) the situation where the foot put pressure on side part (e-4) heel part (e-5) frontal part of the insole.

Finally, to test the real-world applications of the fabricated sensors, four different experiments were conducted. First, a female subject was asked to integrate the sensor (Type-1) on her knee and tasked with specific movements. The results are shown in (Fig. 15a) and (Fig. 15d1). Then the same procedure was used to test the same sensor on the elbow (Fig. 15b) and (Fig. 15d2). The subject was asked to do specific movements on her elbow to demonstrate another application of these sensors as a measurement system in physical therapies or performance measurements. Finally, to conclude a set of complete movements, the same sensor was placed on the subject's wrist and asked to follow a set of motions which included the twist and bending motion, and the results are shown in (Fig. 15c). As shown in (Fig. 15a–c), the proposed sensor can adequately measure the movement of the joints like the knee, elbow, and wrist. It can also detect complex movements like twisting the wrist or neck. These results can assure that the proposed sensor has enough potential to be used in real-world applications like rehabilitation, wearable devices, soft robotics, smart clothing, gait analysis, motion capture, AR/VR, etc.

At last, one of the distinctive features of fabricated sensors was tested. The novel fabrication method used in this paper made it possible to fabricate three-dimensional channels that translate to another degree of freedom in sensing applications. As shown in (Fig. 5c) it is plausible to produce various gradients to grasp a better sense of direction in these sensors and design them for specific purposes. Through this concept, a whole active foot insole was fabricated using the same material integrating sixteen 3D sensors. (Trapezoidal by side view) (Fig. 15e1). These sensors can measure the amount of pressure applied to them due to their 3D structure. Furthermore, because of the designed gradients in height throughout the foot insole plane, it is feasible to discern the points of pressure more accurately, all due to the extraordinary capabilities that the new fabrication method brought with it. The diagram shown in (Fig. 15e2) until (Fig. 15e5) shows the practical results of using the foot insole by a female subject. The excellent pressure pinpoint ability of the active foot insole can be utilized to screen specific problems such as flat feet and improper walking forms in children. Figure 15e2 shows the pressure gradient graph when the foot puts pressure on all parts of the insole. Figure 15e3 depicts the situation where the foot puts more pressure on the side of the insole, and also (Fig. 15e4) illustrates the condition when the foot presses against the ankle. Finally, (Fig. 15e5) shows a posture in which the foot presses against the forefoot.

## Conclusion

This paper proposes a novel approach for a fully 3D, microfluidic-oriented, gel-based, and low-cost resistive soft sensor. By the proposed method, we can fabricate various sensors that could quantify all of the stretch, twist, and pressure. To show the proposed sensors' functionality, the fundamental physics behind this resistive sensor was first investigated, and then one of the sensors was simulated using FEM simulations. Then, a set of the designed experimental tests was done. Experimental tests showed that the sensors are linear and accurate when tested with mechanical signals, like Sinusoidal, Ramp, Pulse, and PRBS. The sensor showed a multi-functional response to stretch, twist, and pressure. However, to distinguish and measure all the stretch, twist, and pressure applied at once, more complex data analysis like machine learning and deep learning algorithms are required, which will be investigated thoroughly in future researches. Also, by a 4-h experiment, the durability of the proposed sensors was proven. Finally, the sensor was put through its paces on a female test subject's knee, elbow, and wrist. Also, to evaluate the pressure functionality of the sensor, a complete 3D active foot insole was fabricated and then tested. The results were displayed using a gradient graph of the foot. These results assure us that the proposed sensor has enough potential to be used in real-world applications like rehabilitation, wearable devices, soft robotics, smart clothing, gait analysis, motion capture, AR/VR, and many others.

## Data Availability

The datasets used and analyzed during the current study are available from the corresponding author upon reasonable request.
